# Salt-Reduced Fish Sauce Produced under Pressurized Carbon Dioxide Treatment Using *Sardinops melanostictus*, *Trachurus japonicus*, *Konosirus punctatus*, *Odontamblyopus lacepedii*, Their Collective Mixture, and Unused Fish Mixture

**DOI:** 10.3390/foods13172646

**Published:** 2024-08-23

**Authors:** Johma Tagawa, Mikihide Demura, Seiji Noma

**Affiliations:** 1 Graduate School of Agriculture, Saga University, Saga 840-8502, Japan; 2 Faculty of Agriculture, College of Natural Sciences, Institute of Education and Research, Saga University, Saga 840-8502, Japan; 3 The United Graduate School of Agricultural Sciences, Kagoshima University, Kagoshima 890-0065, Japan

**Keywords:** fish sauce, carbon dioxide, reduced salt, odor, taste, commonality, unused fish

## Abstract

Fish sauce is produced at high salt concentrations (>20%) to inhibit the growth of harmful microorganisms. The salt-reduced fish sauce (10% salt) was prepared under pressurized CO_2_ (pCO_2_) conditions at 30 °C and 5 MPa for 3 months (${FS}_{{CO}_{2}}$), from *Sardinops melanostictus*, *Odontamblyopus lacepedii*, *Trachurus japonicus*, *Konosirus punctatus*, and their collective mixture, as well as unused fish mixture obtained from the Ariake Sea in Japan. ${FS}_{{CO}_{2}}$ exhibited significantly better microbial quality and free amino acid content, lighter color, standardized odor (dashi-like odor), and umami richness qualities compared to fish sauces prepared using the conventional method (${FS}_{con}$) (20% salt), as previously demonstrated, after a fermentation period of 2 months. Bacterial flora analysis implied that the standardization of odor and umami richness may not be the result of specific microbial metabolism. Even when using previously unused fish, it was possible to produce ${FS}_{{CO}_{2}}$ equivalent to that produced by conventional sardines and other fish. These results indicate that the quality of fish sauce can be improved. The flavor of ${FS}_{{CO}_{2}}$ became similar regardless of the type of fish and fermentation period using pCO_2_ during fermentation, leading to the effective utilization of unutilized fish as a resource for high-quality salt-reduced fish sauce.

## 1. Introduction

Fish sauce is a liquid seasoning made by marinating fish in a high salt concentration and allowing it to digest. It is produced through protein hydrolysis by the action of endogenous proteases in the fish body and the metabolism of contaminating bacteria, which contribute to the formation of a unique flavor during the fermentation process [1]. High salt concentration is essential to inhibit the growth of spoilage bacteria that produce unfavorable flavors and amines in fish sauce [2]. However, consumer demand for salt reduction is increasing, and high salt content hinders the decomposition of fish meat. Salt reduction has been attempted through electrodialysis and the addition of acetic acid [3,4]. However, these methods do not address the retarded decomposition rate of fish meat, despite meeting consumer demand.

We attempted to reduce salt concentration by applying pressurized CO_2_ (pCO_2_) during fermentation. Both acidic and anaerobic conditions can be created under pCO_2_ treatment, which is expected to inhibit the growth of aerobic mesophilic bacteria and prevent the oxidation of fish sauce components. The use of pCO_2_ eliminates the need for CO_2_ removal after fermentation because the CO_2_ dissolved in fish sauce is naturally released after depressurization. Noma et al. [5] demonstrated that reduced-salt (10% of the final salt concentration) sardine fish sauce could be prepared under pCO_2_ treatment (30 °C and 1–5 MPa for 6 months). The resulting fish sauce had improved qualities, including increased free amino acid content, brightened color, and suppressed characteristic mustiness, compared to the conventional production method under atmospheric pressure with 20% NaCl concentration. Furthermore, Tagawa et al. [6] prepared a fish sauce from *Sardinops melanostictus*, *Odontamblyopus lacepedii*, *Trachurus japonicus*, *Konosirus punctatus*, and their collective mixture under pCO_2_ at 30 °C and 5 MPa for 2 months. The quality of the fish sauces (${FS}_{{CO}_{2}}$) similarly improved, regardless of the fish species used as the raw material. Interestingly, the flavors (odor and taste) of ${FS}_{{CO}_{2}}$ tended to be similar to each other. These findings suggest that fish sauce with a standardized flavor can be prepared from unused marine resources. It is unclear whether the advantages of fermentation under CO_2_ and similarity of flavor among various kinds of fish can also be ensured using a fermentation period longer than 2 months. Therefore, as a continuation of previous experiments, the improvement in fish sauce quality that we observed at 2 months was tested using a longer fermentation period of 3 months. In addition, we have not yet determined whether bacteria produce common flavors in ${FS}_{{CO}_{2}}$.

Sardines are the main raw material used in fish sauces worldwide. However, reliance on a specific species of sardine as a raw material may lead to its depletion. In addition, a non-negligible amount of catch is wasted at sea immediately after harvesting, which increases fishing costs. If fish sauce of similar quality to sardines can be produced even if the raw material is a mixture of unused fish, fishery resources can be conserved and fishing costs reduced using these fish as a raw material for fish sauce without having to sort them by species after harvesting. Many fish species have been identified in the Ariake Sea in Japan; however, many remain underutilized because of their low visibility. Therefore, these fish species can be used as raw materials for fish sauce production.

Therefore, to further develop the study of ${FS}_{{CO}_{2}}$ [6], ${FS}_{{CO}_{2}}$ was prepared from *Sardinops melanostictus*, *Odontamblyopus lacepedii*, *Trachurus japonicus*, *Konosirus punctatus*, their collective mixture for a prolonged fermentation period of three months, and its quality was analyzed. In addition, we prepared ${FS}_{{CO}_{2}}$ from unused fish caught in the Ariake Sea, Japan, and compared its quality with that of sardine ${FS}_{{CO}_{2}}$ to explore the possibility of using unused fish as raw material for ${FS}_{{CO}_{2}}$ in terms of stable quality and flavor. The results obtained in the present study will provide an opportunity to valorize underutilized fish as high-quality fish sauce.

## 2. Materials and Methods

### 2.1. Preparation of Fish Sauces

*Sardinops melanostictus*, *Odontamblyopus lacepedii*, *Trachurus japonicus*, and *Konosirus punctatus* used in this study were purchased from a local supermarket or fresh fish store on the day the fish sauce preparation was started (Thursday, 4 March 2021), and brought back to the laboratory while being kept on ice. In another set of experiments, fish species that are unused and/or rarely used because of their low commercial value, caught in the Ariake Sea (unused fish), were acquired from a fisherman on the day of fish sauce production (Wednesday, 13 July 2022). *S. melanostictus* was repurchased on the same day to prepare fish sauce for comparison with the unused fish sauce. The weights and lengths of the fish used in this study are listed in Table 1 and Table 2, respectively. Fish sauce was produced according to the method described by Tagawa et al. [5]. Fish sauce using the unused fish was prepared by mixing each fish species according to their composition ratio at the time of fishing in the Ariake Sea (*Pennahia argentata*:*Cynoglossus abbreviatus*:*Pampus puntatissimus*:*O. lacepedii* = 5:2:2:1 by weight). The fish mixture prepared in the experiment started on 4 March 2021, and 13 July 2022, and was referred to as “collective mixture” and “unused fish mixture”, respectively.

### 2.2. Fish Sauce Characterization

The degree of fish meat decomposition in the fish sauce mashes was visually observed, and the odor impression was evaluated.

### 2.3. Viable Mesophilic Bacteria Count

The fish sauce moromi was coarsely filtered through a drainage net and centrifuged (1000× *g*, 15 min, 4 °C). A volume of 100 μL of fish sauce sample was serially diluted in 0.85% NaCl solution, plated on Tryptic Soy agar (TSA; Difco, Detroit, MI, USA), and incubated at 30 °C for 2 days. Colonies formed on the plate (10–1000 colonies/plate) were counted, and viable counts were expressed as colony-forming units (CFU/mL).

### 2.4. Biogenic Amine Content

The proteins and fats contained in the fish sauces were removed according to the method described by Noma et al. [5] after rough filtration and centrifugation. The biogenic amine contents of the resulting fish sauces were analyzed using a pre-column derivatization method with dansyl chloride. Briefly, amines in the fish sauce samples and standard amines derivatized with dansyl chloride (Tokyo Chemical Industry Co., Ltd., Tokyo, Japan) were separated by isocratic elution using 75% acetonitrile (Sigma-Aldrich Co. LLC, St. Louis, MO, USA) as the mobile phase on an Intersil ODS-SP column (GL-science Inc, Tokyo, Japan) at a flow rate of 1.0 mL/min and a temperature of 40 °C, and detected using a UV detector at a wavelength of 254 nm. The concentration of each amine in the fish sauce samples was determined by comparing the areas of the peaks with retention times matching those of the standard sample. The analysis was performed with an HPLC system, an L-6200 intelligent pump, an L-5090 degasser, and an L-2400 UV detector (Hitachi High-Technologies Co., Tokyo, Japan).

### 2.5. Free Amino Acid Content

The free amino acid composition of fish sauces without proteins and fats was analyzed using the method described by Noma et al. [5]. Briefly, free amino acids in fish sauces and standard amino acid mixture (FUJIFILM Wako Pure Chemical, Osaka, Japan) were derivatized using phenylisothiocyanate (PTC, FUJIFILM Wako, Osaka, Japan) and analyzed using the HPLC system with Wakopak^®^ Wakosil-PTC (4.0 mm × 250 mm, FUJIFILM Wako) under a gradient condition from 0% (0 min) to 70% (15 min) of solvent B at a flow rate of 1.0 mL/min at 40 °C, and detection was performed at 254 nm. Eluents A and B were specifically designed for analysis using a column (FUJIFILM Wako). The concentration of free amino acids was determined by comparing the peak areas in fish sauces with a standard amino acid mixture.

### 2.6. Organic Acid Analysis

After defatting and deproteinizing the fish sauce, 25 µL of the fish sauce samples were analyzed with the Nexera XR organic acid analysis system (Shimadzu, Kyoto, Japan) according to the manufacturer’s protocol. The concentration of each organic acid in the fish sauce samples was calculated by comparing the peak areas with those of a standard organic acid mixture.

### 2.7. Sensory Evaluation

The odor of the fish sauce was evaluated using a two-point test. Ten non-trained panelists (four males and six females, ages 21–24) participated in the test. The test was conducted to confirm whether an untrained panel representing typical consumers could distinguish between ${FS}_{con}$ and ${FS}_{{CO}_{2}}$. Two milliliters of the fish sauce sample was placed in a test tube and covered with aluminum foil so that the type of sample could not be determined visually from its appearance. Each test tube containing a fish sauce sample was randomly assigned a three-digit number, and the samples were allowed to stand at room temperature for 30 min before sensory testing. The panels were asked to select the one fish sauce with stronger putrefactive odor, fishy smell, rancid odor, shore scent, soup stock-like scent, and preferable odor when comparing between ${FS}_{{CO}_{2}}$ and ${FS}_{con}$ prepared from each fish species. A binomial distribution with *p* = 1/2 was used for the significant difference test.

### 2.8. Analysis of Volatile Compounds

Volatile compounds in the fish sauce were analyzed by solid-phase microextraction-gas chromatography-mass spectrometry (SPME-GC-MS) according to Tagawa et al. (2022) [6]. In brief, fish sauce samples in glass sample bottles were absorbed at 37 °C for 40 min on SPME fiber (divinylbenzene dispersion/dimethylsiloxane, SUPELCO, MilliporeSigma, Darmstadt, Germany) and desorbed at 210 °C for 10 min within the GC-MS (GC-MS-QP2010, Shimadzu, Kyoto, Japan) for analysis. DB-WAX (30 m × 0.25 mm i.d. × 0.25 µm film thickness) (Agilent Technologies, Santa Clara, CA, USA) was used for the separation of the volatiles. After GC-MS, a similarity score was calculated for the detected peaks (peak area > 100,000) according to the following formula, where *n*_FS_ and *m*_FS_ represent the number of peaks in each fish sauce and the number of fish sauces containing the compound (*m*_FS_ takes the value 1–5), respectively:

(1) $$Similarity\;score=\frac{\sum {(n}_{FS}\times m_{FS})}{\sum n_{FS}\;at\;m_{FS}}$$

### 2.9. Taste Evaluation Using a Taste Sensor

The taste of each fish sauce was evaluated using a TS-5000Z system (Intelligent Sensor Technology, Inc., Kanagawa, Japan).

### 2.10. Bacterial Flora Analysis by Next-Generation Sequencing

Total DNA was extracted from the fish sauce using an extraction kit (DNA Suisui-E without skim milk, RIZO Inc., Ibaraki, Japan) according to the manufacturer’s protocol. 16S rRNA (V4) amplicon metagenomic sequencing and determination of operational taxonomic units as relative abundances were performed by Novogen Co., Ltd. (Beijing, China).

### 2.11. Statistical Analysis

Results are expressed as averages of the triplicate measurements ± standard deviation. Principal component analysis (PCA) of free amino acid content and taste was performed using JMP Pro 17.0.0 (JMP Statistical Discovery LLC, Cary, NC, USA). Significant differences in viable count, amine content, and organic acid content were determined using Student’s *t*-test and/or Tukey–Kramer’s method at *p* < 0.05, using Bell Curve for Excel 3.21 (Social Survey Research Information Co., Ltd., Tokyo, Japan).

## 3. Results and Discussion

In this study, fish sauces were prepared from various fish under pCO_2_ conditions for three months. For ${FS}_{{CO}_{2}}$, mesophilic bacteria were not detected. The brighter color and more similar and less distinctive flavor, regardless of the raw material, were characteristics of the ${FS}_{{CO}_{2}}$. These improved qualities were equal to or greater than those of 2-month ${FS}_{{CO}_{2}}$ [6], suggesting that extending the fermentation period was favorable for improving the quality of ${FS}_{{CO}_{2}}$. Interestingly, the quality of ${FS}_{{CO}_{2}}$ prepared from the unused fish mixture was comparable to that of sardines, the most popular raw material for fish sauce.

### 3.1. Characterization of ${FS}_{{CO}_{2}}$

#### 3.1.1. Appearance, Odor Impressions, and Color

**Figure 1:**
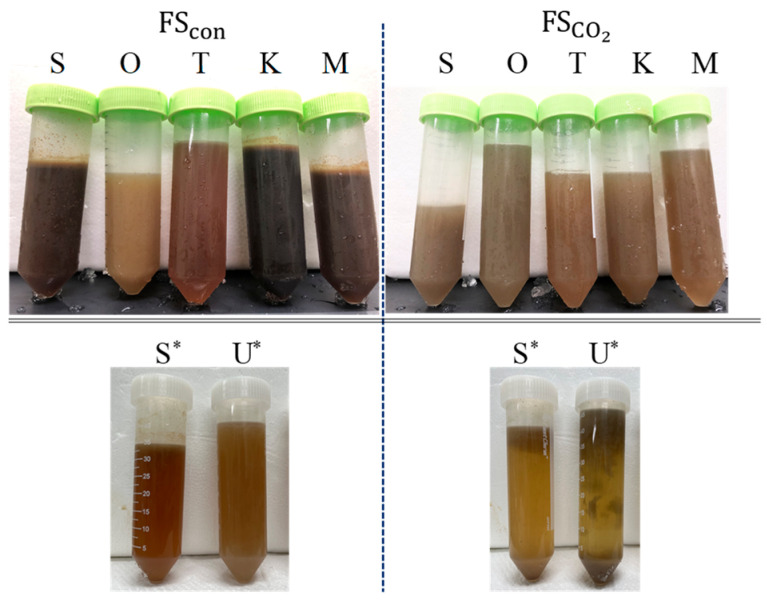
Appearances of fish sauce mashes. S, *S. melanostictus*; O, *O. lacepedii*; T, *T. japonicus*; K, *K. punctatus*; M, collective mixture; S*, *S. melanostictus*; U*, unused fish mixture. * The set of experiments comparing ${FS}_{{CO}_{2}}$ prepared from *S. melanostictus* and an unused fish mixture.

The appearance and odor impressions of ${FS}_{{CO}_{2}}$ and ${FS}_{con}$ mashes are summarized in Table 3. The fish sauce mash was not subjected to the conventional residue separation procedures used in the preparation of fish sauce, such as filtration, and thus exhibited pronounced turbidity. This observation suggests that the fish may still be undergoing decomposition. Fish meat decomposition was better in ${FS}_{{CO}_{2}}$ mash than in ${FS}_{con}$ mash. ${FS}_{{CO}_{2}}$ mash commonly had a soup stock-like odor, although ${FS}_{{CO}_{2}}$ prepared from *O. lacepedi* has a characteristic odor. However, the characteristic fishy and pungent odor impressions of each raw material were observed in ${FS}_{con}$ mashes. The odor tended to be more average in both the ${FS}_{con}$ and the ${FS}_{con}$ mashes when the collective mixture was used as the raw material. As shown in Figure 1, the color of ${FS}_{{CO}_{2}}$ was lighter than ${FS}_{con}$ regardless of fish species. Color formation during fish sauce fermentation can be attributed to the Maillard reaction. The Maillard reaction is initiated by the formation of a Schiff base by the reaction between amino and carbonyl compounds and produces melanoidin at the end of the reaction series, resulting in browning. ${FS}_{{CO}_{2}}$ was prepared under CO_2_-generated anaerobic conditions that suppressed aldehyde formation via lipid oxidation. Horikawa [7] reported that in the middle stage of the Maillard reaction, the Amadori rearrangement proceeds relatively close to neutral. pCO_2_ caused the acidification of the fish sauce, which retarded the progress of the reactions after the middle stage.

#### 3.1.2. Microbial Quality

**Figure 2:**
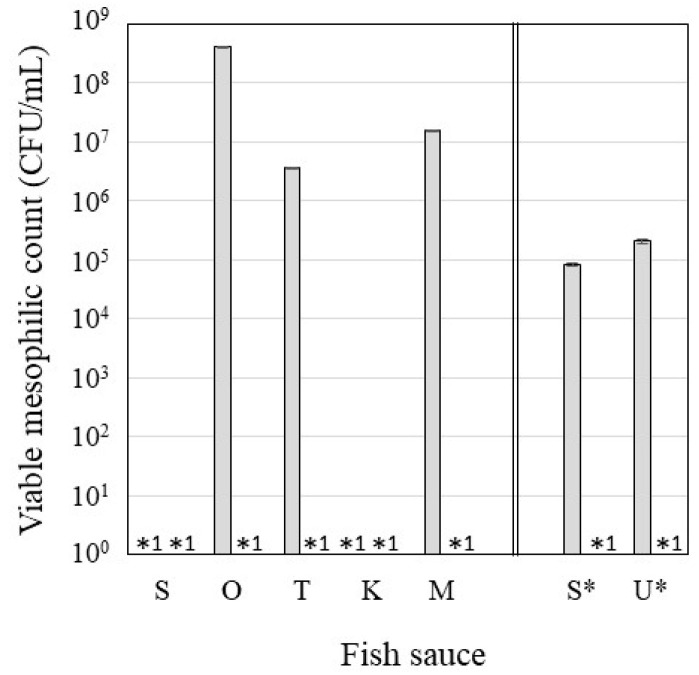
Viable mesophilic counts of each fish sauce. S, *S. melanostictus*; O, *O. lacepedii*; T, *T. japonicus*; K, *K. punctatus*; M, collective mixture; S*, *S. melanostictus*; U*, unused fish mixture. Gray bars indicate ${FS}_{con}$. *^1^ Not detected. * Set of experiments comparing ${FS}_{{CO}_{2}}$ prepared from *S. melanostictus* (S*) and an unused fish mixture (U*).

Lopetchant et al. [8] reported that a salinity of more than 20% inhibited the growth of bacteria to an extent that did not adversely affect the quality of fish sauce. Figure 2 shows the viable mesophilic bacterial counts in each fish sauce. The counts in ${FS}_{con}$ prepared from *O. lacepedii*, *T. japonicus*, or their collective mixtures exceeded 10^6^ CFU/mL. In an experiment comparing *S. melanostictus* and unused fish mixture, mesophilic bacteria were detected in both ${FS}_{con}.$ Therefore, 20% salinity was insufficient to inhibit mesophilic bacterial growth. Wang et al. [9] reported that the microbial flora in fish sauce changed dramatically during each fermentation period. In addition, bacterial flora and fish vary greatly with the season [10,11]. The growth inhibition effect of 20% salinity was not consistent with that observed in our previous report, with a shorter fermentation period (2 months) and the season at the start of fermentation [6].

${FS}_{{CO}_{2}}$ did not contain any detectable mesophilic bacteria, despite 10% salinity. The difference in viable counts between ${FS}_{con}$ and ${FS}_{{CO}_{2}}$ was significant for *O. lacepedii*, *T. japonicus*, and their mixture. Mesophilic bacteria in ${FS}_{{CO}_{2}}$ may be inactivated under acidic pH, anaerobicity, and high pressure caused by pCO_2_ [12]. Higher CO_2_ pressure increases the permeability and fluidity of bacterial membranes, causing a decrease in phosphoglycerides relative to phosphatidylethanolamine, which may be essential for microbial growth, and its reduction may lead to microbial inactivation. Changes in the charge balance on the membrane surface owing to a decrease in the pH of the membrane may also cause inactivation. Furthermore, pCO_2_ causes destruction of cell surface or cellular tissue [13,14,15] and inactivation of microbial enzymes [16]. Anaerobic conditions were generated during the ${FS}_{{CO}_{2}}$ fermentation process as CO_2_ was dissolved in the salt solution with fish and filled the pressure-resistant vessel. As many mesophilic bacteria grow aerobically, anaerobic conditions under pCO_2_ conditions may inhibit their growth. Mesophilic bacteria were not detected regardless of the fish species in ${FS}_{{CO}_{2}}$. This result is in agreement with Tagawa et al. [6], indicating that factors such as the length of the fermentation period and fishing season have no effect on the suppression of mesophilic bacteria in ${FS}_{{CO}_{2}}$, and the pCO_2_ condition is suitable for producing fish sauce with more stable quality.

Biogenic amines are commonly present in fermented foods [17,18]. According to the Codex standards [19], the concentration of histamine should not exceed 400 ppm, as it has the greatest effects on the human body, including nausea and headache among the amines [20,21]. Histamine production in fish is primarily attributed to histidine decarboxylase enzymes of histamine-producing bacteria [22]. High concentrations of tyramine cause headaches and other physiological effects at high concentrations [22,23,24]. Putrescine, cadaverine, and spermidine do not have physiological effects by themselves, but they enhance the physiological effects of histamine and tyramine or inhibit enzymes that decompose histamine [25,26]. Table 4 presents the biogenic amine content of each fish sauce sample. The average contents of putrescine and cadaverine in ${FS}_{con}$ and ${FS}_{{CO}_{2}}$ were significantly (*p* < 0.05) higher than those of the other three amines. The mean putrescine and cadaverine levels were also higher in ${FS}_{{CO}_{2}}$ than in ${FS}_{con}$ Histamine was detected at levels below the Codex specification (40 mg/mL) in both ${FS}_{con}$ and ${FS}_{{CO}_{2}}$. Uehara et al. [27] reported that histamine production was higher when fish with internal organs underwent fermentation. Noma et al. (2020) reported that histamine was not detected in ${FS}_{con}$ and ${FS}_{{CO}_{2}}$ after 6 months of fermentation of sardines with the removal of internal organs under pCO_2_ [5].

### 3.2. Flavor Quality of ${FS}_{{CO}_{2}}$

#### 3.2.1. Odor

**Figure 3:**
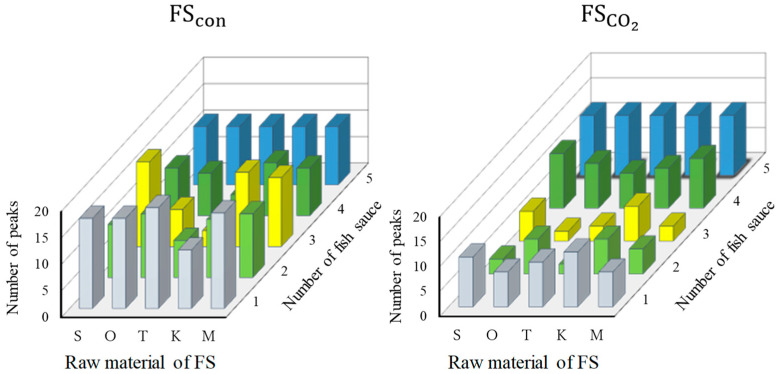
The numbers of peaks commonly observed in each fish sauce. S, *S. melanostictus*; O, *O. lacepedii*; T, *T. japonicus*; K, *K. punctatus*; M, collective mixture.

The volatile compounds in each fish sauce were analyzed using GC-MS, and the obtained peaks with areas larger than 100,000 were compared. For ${FS}_{con}$, the numbers of *S. melanostictus*, *O. lacepedii*, *T. japonicus*, *K. punctatus*, and their collective mixture were 63, 55, 44, 57, and 63, respectively. For ${FS}_{{CO}_{2}}$, the numbers of peaks were 42, 37, 43, 35, and 37 for *S. melanostictus*, *O. lacepedii*, *T. japonicus*, *K. punctatus*, and their collective mixture, respectively. The numbers of peaks commonly observed for each fish sauce are shown in Figure 3. For example, 16 peaks observed in ${FS}_{con}$ prepared from *S. melanostictus* were also detected in the three types of ${FS}_{con}$. For ${FS}_{con}$ prepared from S, O, T, and M, the number of volatile compounds contained in the sample was greater than 17. The peaks common to all ${FS}_{{CO}_{2}}$ were not specific to ${FS}_{{CO}_{2}}$, and were also common to all or some of the ${FS}_{con}$. The ratio of the number of peaks specific to one fish species or common to two fish species was 47.5% in ${FS}_{con}$, which was higher than that in ${FS}_{{CO}_{2}}$ (35.0%). The ratio of the peaks specific to ${FS}_{con}$ was calculated as 66.3%. Therefore, the results suggest that ${FS}_{con}$ contains fish-specific odor components, while ${FS}_{{CO}_{2}}$ enables the production of fish sauce with a reduced fish-specific odor.

The peak similarity score, which indicates how many fish species the volatile compounds present in each ${FS}_{{CO}_{2}}$ and ${FS}_{con}$ were common to on average, was calculated. Fish sauces with higher scores indicated higher similarity in volatile compounds among fish sauces. The score for ${FS}_{{CO}_{2}}$ was 3.27 and that for ${FS}_{con}$ was 2.77. Thus, the fish sauce preparation methods with higher scores indicated a higher similarity of volatile compounds among the fish sauces, indicating that the volatile compound profiles of ${FS}_{{CO}_{2}}$ were more similar than those of ${FS}_{con}$. This result was consistent with the observation that the unique odor of the fish species was suppressed, and the common odor of fish sauce was a soup stock-like odor in ${FS}_{{CO}_{2}}$ (Table 3). A 16% increase in the percentage of peaks commonly present in ${FS}_{{CO}_{2}}$ was observed in the unused fish mixture, indicating that the types of volatile compounds tended to be similar. The odor characteristic of ${FS}_{{CO}_{2}}$ showed a decreased fish-specific odor and an increased soup stock-like odor. This result is consistent with those observed in the sensory analysis in this study.

Table 5 shows the odor attributes with significant differences (*p* < 0.05) and significant trends (*p* < 0.11) in ${FS}_{{CO}_{2}}$ by sensory evaluation. The quantification of the odor impressions is shown in Table 3. In ${FS}_{{CO}_{2}}$, putrefactive and rancid odors were reduced, preferred odors were enhanced regardless of the fish species, and the soup stock-like scent tended to be enhanced. In addition, when comparing the *S. melanosticus* and the unused fish mixtures, similar differences were observed. These trends were in agreement with those of some 2-month fish sauces [6], and the longer fermentation period of 3 months in the present study tended to improve the odor of ${FS}_{{CO}_{2}}$. This suggests that a fermentation period of 3 months or longer is preferable for the production of ${FS}_{{CO}_{2}}$ in terms of odor. Furthermore, all panelists distinguished between ${FS}_{{CO}_{2}}$ and ${FS}_{con}$ prepared from *S. melanostictus* and an unused fish mixture. However, more than half of the panelists could not distinguish between ${FS}_{{CO}_{2}}$ prepared from a mixture of *S. melanostictus* and the unused fish mixture. The results of both GC-MS and sensory analyses demonstrated that the ${FS}_{{CO}_{2}}$ exhibited a comparable odor profile, suggesting that pCO₂ can be employed to generate fish sauces with a similar odor profile, even when utilizing unspecified fish as the raw material.

#### 3.2.2. Taste

**Figure 4:**
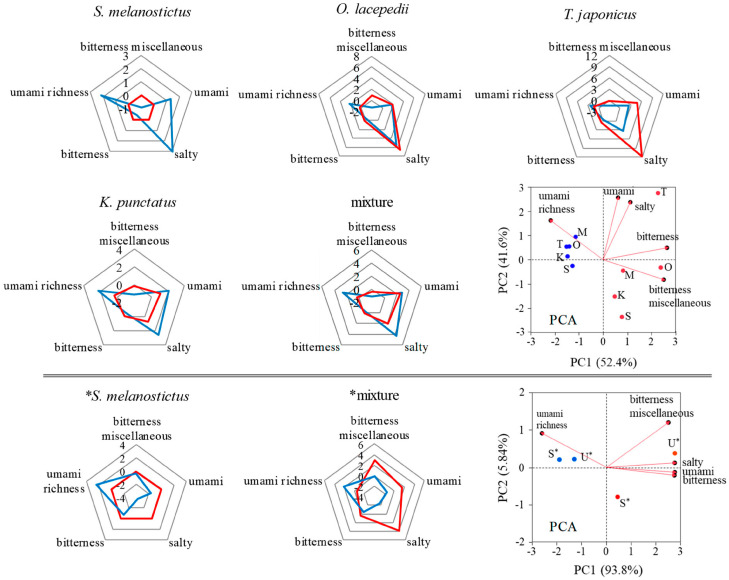
Comparison of tastes detected in ${FS}_{{CO}_{2}}$ and ${FS}_{con}$ with radar charts and their PCA. Blue and red lines/dots in the graphs indicate ${FS}_{{CO}_{2}}$ and ${FS}_{con}$, respectively. Blue and red dots on PCA graphs show ${FS}_{{CO}_{2}}$ and ${FS}_{con}$, respectively. S, *S. melanostictus*; O, *O. lacepedii*; T, *T. japonicus*; K, *K. punctatus*; M, collective mixture; S*, *S. melanostictus*; U*, unused fish mixture. * The set of experiments comparing ${FS}_{{CO}_{2}}$ prepared from *S. melanostictus* (S*) and an unused fish mixture (U*).

The taste analyzers recognized three types of first tastes (salty, umami, bitterness and miscellaneous), and two types of aftertastes (bitter and umami). Figure 4 shows a radar plot comparing the tastes detected in ${FS}_{{CO}_{2}}$ and ${FS}_{con}$. The radar shape varied with ${FS}_{con}$, and the bitterness of miscellaneous tastes commonly decreased in ${FS}_{{CO}_{2}}$.This tendency was more pronounced in 3-month fish sauce compared than in 2-month fish sauce [6]. Figure 4 shows the PCA of the tastes detected in ${FS}_{{CO}_{2}}$ and ${FS}_{con}$. The distribution of ${FS}_{con}$ is scattered, whereas that of ${FS}_{{CO}_{2}}$ is similar. These results suggest that the taste of ${FS}_{con}$ reflects the characteristics of each raw fish, whereas in ${FS}_{{CO}_{2}}$, regardless of the raw fish, the tastes converged to the same family, characterized by enhanced umami richness and reduced bitterness.

Table 6 shows the amount of free amino acids in each fish sauce sample. The total amounts of free amino acids in ${FS}_{{CO}_{2}}$ prepared from *S. melanostictus*, *O. lacepedii, T. japonicus*, *K. punctatus*, and their collective mixture, respectively, were 1.9, 2.2, 1.3, 1.7, and 1.4 times and significantly (*p* < 0.05) higher than those in ${FS}_{con}$. Furthermore, a significant (*p* < 0.05) increase in total free amino acid content was observed in the ${FS}_{{CO}_{2}}$ prepared from the unused fish mixture. Free amino acids are produced by endogenous proteases and the contaminating bacteria [1]. Endogenous acid proteases can effectively hydrolyze fish proteins in a pCO_2_ environment because the number of contaminating bacteria may be reduced under such conditions.

**Figure 5:**
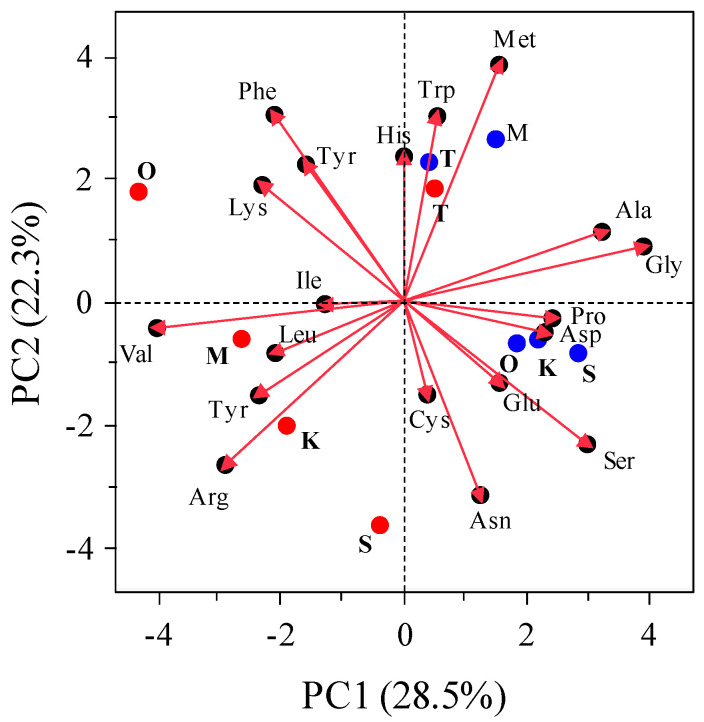
PCA analysis of free amino acid relative composition of fish sauces. Blue and red dots in the graphs indicate ${FS}_{{CO}_{2}}$ and ${FS}_{con}$, respectively. S, *S. melanostictus*; O, *O. lacepedii*; T, *T. japonicus*; K, *K. punctatus*; M, collective mixture.

Therefore, the enrichment of total free amino acid content is a universal phenomenon in ${FS}_{{CO}_{2}}$ regardless of fish type. Figure 5 shows the principal component analysis of the proportions of free amino acids in each fish sauce. The free amino acid composition appeared to be divided into two groups, depending on PC1 between ${FS}_{{CO}_{2}}$ and ${FS}_{con}$. Glutamic acid, a typical umami amino acid, does not always constitute this group. The PCA results indicate that the free amino acid composition did not provide sufficient evidence for enhanced umami taste and umami richness. Imai et al. [28] reported that the bitter taste was not caused mainly by free amino acids but by peptides such as carnosine and glutathione. Kuroda et al. [29] reported that a tripeptide, glutamylvalylglycine, detected in fermented foods such as fish sauce, has umami richness.

**Figure 6:**
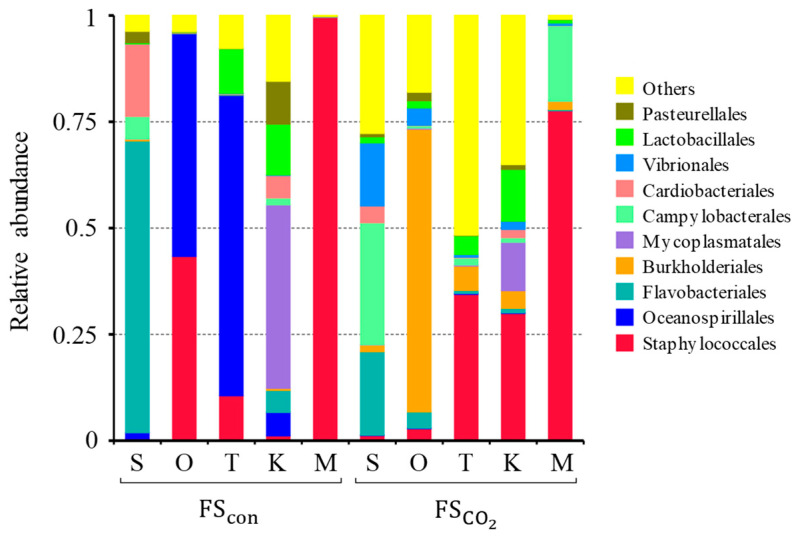
16S rRNA amplicon sequencing analysis and presented as relative ratios of microbiota. S, *S. melanostictus*; O, *O. lacepedii*; T, *T. japonicus*; K, *K. punctatus*; M, collective mixture.

Jung et al. [30] and Ohshima et al. [1] reported that microbial metabolism during fermentation plays an important role in flavor formation in fermented fish-based foods. Wang et al. [31] reported that the peptides in fish sauce are formed by the hydrolysis of fish proteins by microbial proteases. Therefore, the bacterial flora of each fish sauce was analyzed using 16S rRNA amplicon analysis and presented as relative ratios of the bacterial flora (Figure 6). In all raw fish, the bacterial flora changed with the different methods of fish sauce preparation. However, the dominant species in both the ${FS}_{{CO}_{2}}$ and ${FS}_{con}$ collective mixture were staphylococci. The diversity of microflora appeared to increase in CO_2_ fish sauces. The bacterial flora in fish sauce could not be clearly characterized by ${FS}_{{CO}_{2}}$ and ${FS}_{con}$. It is difficult to infer which bacteria contribute to the common flavor observed in ${FS}_{{CO}_{2}}$. The bacterial flora in fish sauce changes during fermentation [8]. The fermented foods contained dead cells, which were also detected using 16S rRNA amplicon analysis. In addition, 16S rRNA amplicon analysis could not quantify the absolute number of viable cells. Therefore, it is necessary to examine viable cells quantitatively and over time to identify the bacterial species that have a significant impact on flavor similarity.

The organic acid composition was similar for all types of ${FS}_{{CO}_{2}}$, and a high lactic acid content was commonly observed in ${FS}_{{CO}_{2}}$ (Table 7). The types of organic acids used varied depending on the raw material used in ${FS}_{con}$. The average lactic acid content of ${FS}_{{CO}_{2}}$ tended to be higher (*p* = 0.07) than that of ${FS}_{con}$. Lactic acid has a peculiar soft acidity and a low-acid smell. Acidity was not detected in ${FS}_{{CO}_{2}}$ (Table 3 and Figure 4), suggesting that the organic acids had no significant effect on the flavor of ${FS}_{{CO}_{2}}$. Lactic acid in fish sauce is produced by lactic acid bacteria or rigorous mortis [32,33]. However, positive evidence that lactic acid bacteria accumulated lactic acid at high concentrations was not obtained from the microflora analysis (Figure 6). In addition, production via the rigor mortis process provides a clear explanation for the difference in lactic acid content between ${FS}_{{CO}_{2}}$ and ${FS}_{con}$ prepared from *O. lacepedii* and *T. japonicus*.

## 4. Conclusions

The present study revealed that pCO_2_ treatment during the fermentation of fish sauces caused significant species-independent quality improvement and flavor similarity, suggesting that a fermentation period of three months or longer is suitable for achieving stable quality improvement compared with two months. However, bacterial flora analyses of fish sauces after preparation could not predict which bacteria were responsible for the similarity in flavor. Further analysis of the microbial flora over time is needed to link microorganisms and flavor formation, leading to a more stable production of ${FS}_{{CO}_{2}}$. In addition, the use of an unused fish mixture in the present study produced a flavor similar to that produced by sardines. These results fully support our future vision for the use of CO_2_ in fish sauce production, as described in our previous paper. Although this study was conducted using unutilized fish harvested in Japan, the problem of unutilized fish is not limited to this country. Therefore, similar experiments using a variety of underutilized fish harvested from more diverse regions are desirable in the future. Furthermore, small-quantity use-up packs, as seen in many kinds of salt-reduced foods, are one solution to ensure the microbial safety of ${FS}_{{CO}_{2}}$ for practical use.

## Figures and Tables

**Table 1:** Average weights and lengths of fishes used for fish sauce production.

	Single Use		Collective Mixture	
	Weight (g)	Length (cm)	Weight (g)	Length (cm)
*S. melanostictus*	98	23	109	24
*O. lacepedii*	30	26	40	29
*T. japonicus*	210	28	189	27
*K. punctatus*	173	25	164	25

**Table 2:** Weights and lengths of fishes (*S. melanosticus* and unused fish mixture) used for each fish sauce preparation.

	Single Use			Unused Fish Mixture	
	Weight (g)	Length (cm)		Weight (g)	Length (cm)
*S. melanosticus*	101.2	22	*P. argentata*	5.12	8.6
			*C. abbreviatus*	6.79	7.1
			*P. punctatissimus*	8.64	12.8
			*O. lacepedii*	15.65	20.7

**Table 3:** Appearances and odor impressions of ${FS}_{{CO}_{2}}$ and ${FS}_{con}$ mashes.

Raw Materials	FS	Decomposition Degree of Fish Meat	Odor
*S. melanostictus*	${FS}_{con}$	Almost completely	thick
	${FS}_{{CO}_{2}}$	Completely	a little savory, soup stock-like, and stronger than the ${FS}_{con}$
*O. lacepedii*	${FS}_{con}$	Incomplete	fishy, soup stock-like, and characteristic smell of *O. lacepedii*
	${FS}_{{CO}_{2}}$	More than the ${FS}_{con}$	a little savory, soup stock-like, and light
*T. japonicus*	${FS}_{con}$	Completely	fishy, a little soup stock-like, and pungent
	${FS}_{{CO}_{2}}$	Almost completely	savory, soup stock-like, stronger than the ${FS}_{con}$
*K. punctatus*	${FS}_{con}$	Almost completely	${FS}_{con}$ of *S. melanostictus*-like, a little savory, and characteristic smell of *K. punctatus*
	${FS}_{{CO}_{2}}$	Almost completely	a little savory, stronger than the ${FS}_{con}$, and light
Collective mixture	${FS}_{con}$	Almost completely	mixed odor of the 4 kinds of fishes
	${FS}_{{CO}_{2}}$	Almost completely	mixed odor of the 4 kinds of fishes, light, savoy, a little soup stock-like
* *S. melanostictus*	${FS}_{con}$		soup stock-like
	${FS}_{{CO}_{2}}$	Almost	soup stock-like
* Unused fish mixture	${FS}_{con}$		sour and pungent
	${FS}_{{CO}_{2}}$	Almost completely	soup stock-like, shore-like, and a little rancid

* The descriptions below the double lines are for the set of experiments comparing ${FS}_{{CO}_{2}}$ prepared from *S. melanostictus* and an unused fish mixture.

**Table 4:** Biogenic amine content (mg/100 mL) of each fish sauce.

	${FS}_{con}$					${FS}_{{CO}_{2}}$					${FS}_{con}$		${FS}_{{CO}_{2}}$	
	**S**	**O**	**T**	**K**	**M**	**S**	**O**	**T**	**K**	**M**	S*	U*	S*	U*
Putrescine	43	35	37	42	43	57	76	73	59	62	114	100	97	90
Cadaverine	19	12	14	12	7	39	38	60	37	57	31	26	52	44
Histamine	12	6	2	0	0	4	0	5	5	7	0	0	0	0
Tyramine	4	2	2	0	0	2	2	4	1	1	1	0	0	0
Spermidine	8	7	0	0	0	3	2	7	3	4	10	10	14	14

S, *S. melanostictus*; O, *O. lacepedii*; T, *T. japonicus*; K, *K. punctatus*; M, collective mixture; S*, *S. melanostictus*; U*, an unused fish mixture. * The set of experiments comparing ${FS}_{{CO}_{2}}$ prepared from *S. melanostictus* (S*) and an unused fish mixture (U*).

**Table 5:** Odor attributes for which a significant difference or significant tendency was observed in ${FS}_{{CO}_{2}}$.

	Reduced			Enhanced		
Odor Attribute	Putrefactive	Fishy	Rancid	Shores Scent	Soup Stock-Like	Preferred
*p* < 0.05	S, O, T, K, M	M	S, T, K, M	-	O, T, K, M	S, O, T, K, M
*p* < 0.11	-	-	O	S, O, T, K	S	-
*p* < 0.05	S*, U*	U*	U*	-	S*, U*	U*
*p* < 0.11	-	S*	S*	-	-	S*

S, *S. melanostictus*; O, *O. lacepedii*; T, *T. japonicus*; K, *K. punctatus*; M, collective mixture; S*, *S. melanostictus*; U*, unused fish mixture. * The set of experiments comparing ${FS}_{{CO}_{2}}$ prepared from *S. melanostictus* (S*) and an unused fish mixture (U*).

**Table 6:** Free amino acid content (mg/100 mL) of each fish sauce.

	${FS}_{con}$					${FS}_{{CO}_{2}}$					${FS}_{con}$		${FS}_{{CO}_{2}}$	
	S	O	T	K	M	S	O	T	K	M	S*	U*	S*	U*
Gly	67	32	130	44	49	224	153	183	133	150	0	111	80	78
Ala	200	116	374	131	150	425	351	430	329	338	312	265	286	222
Val	116	107	160	126	148	200	162	208	163	175	231	196	225	159
Leu	227	165	305	230	306	380	385	470	336	363	275	253	363	263
Ile	133	91	192	149	189	233	193	285	211	246	196	174	270	158
Ser	86	0	75	71	8	141	137	88	100	89	0	0	116	125
Thr	80	71	131	82	100	150	153	167	118	151	163	172	174	171
Cys	32	7	79	49	73	72	46	36	73	51	69	0	151	0
Met	22	62	167	30	72	132	137	176	145	191	126	111	151	110
Phe	8	52	68	35	42	56	11	65	51	83	137	144	175	147
Tyr	99	75	88	76	84	81	163	109	86	100	163	63	90	90
Trp	81	77	163	109	146	197	182	247	179	220	- **	-	-	-
Pro	58	0	0	81	76	160	118	186	174	222	87	0	209	116
Asn	76	0	0	74	0	173	0	0	122	0	-	-	-	-
Asp	40	28	77	40	74	101	130	70	116	91	118	133	90	90
Glu	68	42	59	41	63	145	159	79	73	105	71	118	175	140
Lys	56	239	165	26	121	154	189	164	137	129	447	214	215	165
Arg	305	187	343	307	437	331	323	313	355	229	0	200	228	201
His	96	67	206	127	154	156	132	298	178	288	284	0	170	211
Total	1850	1418	2782	1828	2292	3511	3124	3574	3079	3221	2679	2154	3168	2446

S, *S. melanostictus*; O, *O. lacepedii*; T, *T. japonicus*; K, *K. punctatus*; M, collective mixture; S*, *S. melanostictus*; U*, unused fish mixture. * The set of experiments comparing ${FS}_{{CO}_{2}}$ prepared from *S. melanostictus* (S*) and an unused fish mixture (U*). ** Not determined.

**Table 7:** Organic acid content (mg/100 mL) of each fish sauce.

	${FS}_{con}$					${FS}_{{CO}_{2}}$				
	S	O	T	K	M	S	O	T	K	M
Citric	3.3	12.9	1.3	6.9	2.6	5.6	1.7	3.6	12.2	10.9
Pyruvic	4.0	3.0	12.9	6.9	1.3	1.7	0.0	4.0	0.0	0.0
Malic	6.6	1.0	0.0	7.3	5.6	8.3	3.0	2.6	9.2	12.5
Succinic	4.6	3.0	0.0	3.3	30.7	6.9	3.6	3.6	2.0	19.5
Lactic	344	2.6	3.3	535	46.2	345	172	738	541	865
Formic	18.2	0.0	0.0	14.9	1.7	1.7	1.3	2.6	3.3	2.3
Acetic	11.2	259	1.7	15.8	352	6.9	4.3	15.8	5.6	36.6
Butyric	0.0	0.3	0.0	0.3	0.0	0.0	0.0	0.0	0.0	0.0
Isovaleric	14.5	21.1	30.0	28.4	45.9	0.0	0.0	0.0	0.0	0.0

S, S. melanostictus; O, O. lacepedii; T, T. japonicus; K, K. punctatus; M, collective mixture.

## Data Availability

The original contributions presented in the study are included in the article, further inquiries can be directed to the corresponding author.
